# Observations on the Rise and Progress of the Medical Art in the British Empire, with a Prospectus, of a> Bibliographia Medicinæ Britannicæ; and Remarks on the Execution of That Work

**Published:** 1807-05

**Authors:** William Royston

**Affiliations:** Princes Street, Cavendish Square


Medical and Phyfical Journal.

VOL. XVII.] MAY, 1807. [NO. 99.

*Printed for R. PHILLIPS, by W. Thorne, Red Lion Court, Fleet Street, London.*

### *To the Editors of the Medical and Phyfical Journal.*

GENTLEMEN,

IN the Medical Journal for the month of June 1806, you did me the honour to announce a Work upon which I have long been employed. Since that time I have been endeavouring to bring it to a state of correctness and maturity that will, I trust, render it deserving the patronage of a learned liberal profession; and I am induced, from the readiness with which you ever promote views that have for their object the improvement of science generally, and the amelioration of the medical art in particular, to hope you will admit into your publication the following "*Observations*", which are intended to explain the design and scope of my undertaking. The composition of a *Bibliographia Medicæ,* upon the plan laid down in the ensuing *Prospectus,* involves depth of research, difficulty of arrangement, extensive information, and multifarious inquiry sufficient to apologize for my soliciting that assistance from my brethren, the arduous nature of my work demands, and which my endeavour to be useful, perhaps, deserves. I am encouraged to request this assistance by the interest a respectable part of the Faculty take in my design; and the aid I have already received warrants an expectation, that, when my object is more fully known, men of science will not be reluctant to forward its completion. The particular points to which I wish to turn the minds of the Faculty, and on which I feel that information would be peculiarly acceptable to myself, are, an elucidation of the early period of medical history in this country; an account of the Theses written by Englishmen at Foreign Universities soon after the revival of literature; descriptions of scarce books, and M S S.; observations on the first appearance of the *sudor anglicanus, rachitis, syphilis, variola, &c.* in England; and biographical no-

 tices tices of professional men, whose history has escaped the attention, or has not come within the plan, of Freind and Aikin.

Before I close this note, permit me to acknowledge how much I have been indebted, for many heads of inquiry, to a masterly sketch by the late ingenious and learned Dr. Edward Milward.

I am, &c.
WILLIAM ROYSTON.

*Princes Street,*
*Cavendish Square, April,* 7, 1807.

---

OBSERVATIONS *on the Rise and Progress of the Medical Art in the British Empire, with a* PROSPECTUS, *of a* BIBLIOGRAPHIA MEDICINÆ BRITANNICÆ; *and Remarks on the Execution of that* WORK.

ON the Continent of Europe several works have appeared, the object of which has been to give a view of the progress of medical literature, by forming descriptive and scientific catalogues of medical books; or by arranging the materials of those books under distinct heads, to afford the student an exact and expeditious reference to whatever subject his studies required. It is matter of surprize, that scarcely an effort has hitherto been made, to form similar arrangements of the medical literature of England. Certainly, the continental Bibliothecarians have not confined their plans to the books of any individual country, but have attempted to give a view of those of all periods and places. This circumstance has occasioned defects in their stupendous and widely extended designs; and has left the indigenous literature of every country too imperfectly described. Those who are acquainted with the genius and talents of Haller, and the persevering industry of Ploucquet, cannot fail to admire the ability with which their Bibliothecas are executed; yet, it must also be admitted, that with respect to English books, they are always jejune, and often incorrect. In the ten quarto volumes of Ploucquet, the references to English books are too few in number to do any manner of justice to the subject; and whoever examines the Bibliothecas of Haller, those astonishing monuments of human knowledge, as well of human labour, will be surprized to find how little he was acquainted with the works of Englishmen, especially those written in the English language. These observations are not made with a view to depreciate the talents, or under-rate the industry of those writers, but to shew shew that the most profound erudition, and the most unwearied application, are not equal to an exact description of the native literature of a foreign country. It then becomes almost a self-evident proposition, that the Bibliothecas of the indigenous literature of any conntry, should not be left in the hands of a foreigner. Several years have now elapsed, since, impressed with this idea, I began to make collections for a *Bibliographia Medicinæ Britannicæ*, in which I desired and almost hoped to unite the precision of a *Catalogue raisonnée*, the advantages of a concordance of facts and opinions, an historical sketch of the progress of the medical art in Britain, and biographical recollections of writers who have become conspicuous by their theories, the controversies in which they have been engaged, by their having meliorated the practice, or by their discoveries extended the boundaries of medical science.

More than half a century has passed away since an attempt was made to execute a plan, bearing some small resemblance to this. Why that attempt was unsuccessful it becomes proper to inquire, to avoid the causes that occasioned its failure. Among those causes, my inquiry has led to a belief, that the most decisive was a singular apathy that pervaded the study of nature, and the pursuit of physical science. Perhaps this is a more auspicious æra. My hope of success, so far as it regards the design of the work, rests principally on the general diffusion of a spirit of literary investigation that distinguishes the present period; on the desire to elucidate the sources from whence knowledge has arisen, and to trace the current of opinion as it flows from age to age. The *amor patriæ* readily suggests that it would have been grateful to Englishmen, at any period, to have beheld another wreath of intellect twined around the genius of our island; but a concurrence of peculiar circumstances was required to make the public feel, that the science of medicine could add one more ornament to the literary character of a country, already adorned by the immortal works of Shakspeare, Milton, and Newton.

The knowledge, the liberal opinion, however, and even the taste of the present age, will not allow a fastidious rejection of the labours of Harvey, Sydenham, Cullen, Hunter, Monro, Darwin, &c. or consider their works as dishonouring the temple of British worthies. Greece, in her brightest day of mental illumination, would have deified the discoverer of the *circulation of the blood.* It

 would would not be difficult to point out a train of facts that exalt the medical character of England to the summit of the scale of merit; classical learning, science, and elegant literature, have adorned it in no common degree; and the numerous works which bear ample testimony to this deserved panegyric, present a system of anatomical and physiological learning, profound investigation, accurate arrangement, ingenious theory, and rational practice, equal, perhaps superior, to that of any other country.

It seems quite unnecessary to enter into a defence of the subject of these "Observations," or to apologize for bringing it before the public, on any other account than for defects in its execution. The general sense of the utility of compilations of this kind is sufficiently obvious, from the support given to the labored Bibliothecas of the continent, and which the universal approbation of the Profession has sanctioned, as decidedly servicable in scientific investigations. The preceding remarks, it is hoped, may also prove, that the medical literature of England is deserving of a Bibliotheca, similar to those of Haller and Ploucquet. The talents, the erudition, and the industry of these writers, however, only fall to the lot of few; to emulate them, except in one point, would be the boldest presumption. Industry and patient investigation may be commanded. If the utility of the general design be admitted, and the particular application of it to the medical literature of England in some degree justified by these "Observations," little more remains than to give a view of the Outline, and point out the peculiarities in the Plan of the projected *Bibliographia Medicinæ Britannicæ.*

It is intended, in the *Bibliographia Medicinæ Britannicæ,* to give a view of the progress of medical literature in England, by forming a *Catalogue raisonnée* of the medical works of Englishmen, beginning with the earliest printed books, and ending with the year 1800; and by a scientific arrangement of the materials of those works, to afford an easy reference to whatever they contain. Subordinate to this will be given biographical notices of Authors, critical remarks, and occasional practical observations; the whole to be preceded by an Introduction, containing an historical sketch of the progress of the Medical Art in Britain, from the earliest period to the present time.

As the introductory part is intended to be a *coup d'œil,* it will touch, perhaps slightly, though it is hoped with precision, on the state of medicine among the ancient

Britons,

Britons, particularly noticing the practice of it by the Druids; the state of medical science under the Romans, in Britain; under the Saxons, the Danes, and the Normans; on the progress of the art from the Norman conquest to the time of Linacre, and the founding of the College of Physicians; with a particular investigation of the practice of physic and surgery by the monks and other ecclesiastics; and an inquiry into the losses that the science has sustained by the destruction of MSS. at the Reformation. The period succeeding to this will be contained between the founding of the College and the discovery or demonstration of the circulation of the *blood.* Linacre, Caius, Ellyot, Turner, the author of the first English Herbal, Moffit, Goulston, Clowes, Woodhall, &c. adorned this period. These learned and ingenious men made, however, no considerable discoveries, because they were too much devoted to the Galenic theory; and their time was employed either in translating or commenting on the works of their master, or in defending the newly recognised rights of the Regulars against the Empirics. The æra of Harvey, which succeeded to this, is highly interesting; his discovery called forth new views, and infused fresh energies into the human mind. The structure and functions of animal bodies were investigated with unwearied assiduity, and the most profound arcana of Nature almost developed. The discoverer of the circulation, with Glisson, &c. now laid the foundations of an English School of Anatomy, that has since risen into proud pre-eminence. The institution of the Royal Society still further encouraged the cultivation of science; and its great work, the Philosophical Transactions, will claim particular attention, as the immense depositary of facts in every province of natural history. It was in that national work Lower, Needham, Thurston, Mayow, Willis, Ridley, and Boyle, first made the learned world acquainted with their discoveries.

In glancing over the progress of the medical art in the British islands, beside the above points, occasion will be taken to notice, as concisely as the nature of the undertaking admits, a number of other circumstances. Biographical accounts of authors, unnoticed by Freind and Aikin, will claim particular attention; as will alchymy, the rosycrutian visions, the intermixture of astrology with the practice of physic, and various other empyrical delusions. The incorporation of the Surgeons of London, establishment of anatomical and chirurgical lectures at Surgeons

 Hall

Hall; at the College of Physicians; the Harveian oration; the rise and establishment of a medical school at Edinburgh, will not pass unnoticed. The disputes between the Galenists and chemists; the attempt to establish a Society of Chemical Physicians, in opposition to the College; the legal investigation of the claims of the Licentiates; and the endowment of Hospitals in London, Edinburgh, Dublin, and the provincial towns; with the effect they have had in improving the theory and practice of physic and surgery, will also make a part of the inquiry. One of the most interesting portions of the history of medicine, is that which endeavours to extricate the origin of certain diseases from the obscurity in which time has enveloped them. An effort will be made to elucidate the first appearance of *siphilis* in Britain; the *sudor Anglicanus*; *variola*; *rachitis*; contagious catarrh, known under the popular term, Influenza. This affection, from its periodical recurrence, from the extent of its influence, and from the rapidity of its progress, affords one of the most remarkable and curious facts in the history of diseases. The appearance and progress of pestis at various periods, and the opinions respecting it; contagion, with the attempts made to correct or destroy it, and the conduct of the Legislature with regard to those attempts; quarantine; rabies canina; scurvy, its first appearance in the British navy, and the management that has almost effected its annihilation; *chirurgia infusoriæ et transfusoriæ*; the introduction of mercury, and the controversy on the use of crude quicksilver as a panacea; the first use of *cinchona*, and how far and how early the English contributed to investigate its properties; the discovery and history of mineral waters; progress and improvement of surgery; practice of the obstetric art, its gradual improvement, and of the efforts made to retain it in the hands of females; empirical attempts to dissolve the calculus of the human bladder; the countenance those attempts received from the Legislature, and the consequent controversy; the introduction of small pox inoculation; discovery of vaccination, and the disputes concerning their utility.

The Introduction is intended to conclude with a view of the progress of botany, chemistry, electricity, and galvanism, as connected with the art of medicine. These are some of the objects that this Introductory Sketch will embrace; and notwithstanding the subjects of it are so multitudinous, and the views so extensive, it is hoped, with some confidence, that it may be condensed to such a degree, gree, as not to occupy too much space in the Work, and yet be rendered perspicuous by a constant and clear reference to the corresponding Classes and Sections of the *Bibliographia*.

It is proposed to arrange the body of the Work under Classes and Sections, according to the following distribution.

CLASS I.

*Anatomy and Physiology.*

CLASS II.

*Materia Medica.*

In this Class will be comprehended the natural history and preparation of substances used in medicine, medical botany, and pharmacopœias.

CLASS III.

*Theory and Practice of Physic.*

The multifarious materials of this Class will require a subdivision into the subsequent Sections.

SECTION 1. *Fever.* Continued, intermittent, and hectic.

SECTION 2. *Exanthemata.* Variola, varicella, rubeola, scarlatina, erysipelas, miliaria, urticaria, pemphigus, pestis, aptha, and variola vaccina.

SECTION 3. *Diseases of the Brain and Nervous System.* Paralysis, apoplexy, epilepsy, catalepsy, chorea, hysteria, hypochondriasis, oneirodynia, mania, melancholia, trismus, tetanus, &c.

SECTION 4. *Diseases of the Chest, and respiring Organs.* Phthisis pulmonalis, asthma, angina pectoris, catarrh, simple and contagious, pneumonic inflammation, dyspnœa, pertussis, &c.

SECTION 5. *Diseases of the Digesting Organs.* Cholera, diarrhœa, dysentery, obstipatio, dyspepsia, diseases of the mouth and fauces, constricted œsophagus, hemorrhois, scirrhus rectum, inflammatory affections of the viscera, and vermes.

SECTION 6. *Diseases af the Heart, and circulating System.* Aneurism, palpitatio, ossification, polypus, &c.

SECTION 7. *Diseases of the Liver and its appendages.*—Icterus, hepatitis, biliary concretions, &c.

SECTION 8. *Diseases of the Urinary Organs.* Calculus, diabetes, nephritis, &c.

SECTION 9. *Dropsy.*

SECTION 10. *Gout, rheumatism, sciatica.*

SECTION 11. *Syphilis.*

SECTION 12. *Diseases of the Eye, the Ear, and the Organ of Smell.*

SECTION 13. *Natural History, Structure, and Diseases of the Teeth.*

SECTION 14. *Chronic Diseases of the Skin.* Elephantiasis, lepra, psora, herpes, tinea capitis, &c.

SECTION 15. *Scrophula.*

SECTION 16. *Scurvy.*

SECTION 17. *Cancer.*

SECTION 18. *Poisons, Animal, Vegetable, and Mineral.*

CLASS IV.

*Surgery.*

CLASS V.

*Obstetrics, with Diseases of Women and Children.*

CLASS VI.

*Mineral Waters.*

CLASS VII.

*Medical Jurisprudence.*

In this class will be contained Prophylaxis, or the preservation of public and private health; revification and the humane society, various branches of medical police, and the conduct of the faculty, as evidences in courts of justice, &c.

CLASS VIII.

*Histories of Medicine, Medical Biography, Charters and Disputes respecting Privileges, Patent Medicines, and Empiricism.*

CLASS IX.

*Glossaries and Dictionaries.*

CLASS X.

*Nosology.*

CLASS XI.

*Veterinaria.*

CLASS XII.

*Experimental Philosophy.*

Electricity, magnetism, galvanism, meterology and natural history, as far as connected with the theory and practice of medicine, will be comprehended in this class.

CLASS XIII.

*Miscellanea.*

From the multitude of books which treat generally on the subject of medicine, in the form of systems, or as comprehending a variety of subjects in the same volume, and Collections, Reviews, Journals, &c. an extensive Class, with the title Miscellanea, became unavoidable.

Beside

Beside the books specified under the above arrangement, it is intended to attach to each Class or Section, a reference to the loose and fugitive materials found in Transactions, Essays, Journals, &c. with the title of *Collectanea.*

The method to be pursued in describing the books of which the *Bibliographia* is to consist, will involve, first, a description of the volume, comprehending the title, size, place, date, number of pages, plates, and other peculiarities; principes and optimæ editions: secondly, an analytical view of the contents, with critical remarks, practical observations, and biographical notices of the author. An endeavour will also be made, to give the anonymous and pseudonymous productions to their real writers.

Among the objects of a *Prospectus*, the public has been accustomed to consider the time of publication, the form, the extent, and the price of a work, as the most essential. If the author of the *Bibliographia Medicinæ Britannicæ* were able to speak explicitly to all these points, it would give him much satisfaction. He is enabled to state, however, that the historical introduction, and some of the Classes, will be ready for publication in the ensuing season; and, if he is warranted, on due consideration, to publish the volumes separately, a part will then appear.—The form is purposed to be 8vo. and it will go to the extent of four or five volumes.

Should it become a question, why a view of the work has been laid before the public, when it is not sufficiently advanced to answer determinately every inquiry, the Author has only to reply, that this has been done with the prospect of gaining information and instruction from liberal criticism, while his work was yet in a state to be corrected by the illumination that might thus be brought to bear upon it. He has not enough confidence in his own powers, to suppose that his is the best of all possible designs, that his arrangement is sufficiently comprehensive, and perfectly lucid; that he has neither attempted too much, nor effected too little. He has many doubts which he hopes thus to have solved, many wishes for information which he expects thus to have completely gratified.

If on the general structure and disposition of the parts of the *Bibliographia Medicinæ Britannicæ*, he is solicitous to have the opinion and advice of his brethren, he also is anxious to have it understood, that the work will not be a mere catalogue of names; but that it is his decided and determined object, to give in it, as compleat a view of the rise and progress of *British Medicine*, in all its ramifications cations cations and dependencies, as the limited range of his acquirements admit. It is his desire to trace to their source every discovery, to elucidate every theory, and to detail every interesting controversy: to point out hypothetical aberrations of opinion, and impress the rational principles and sound reason, that has restored the misguided intellect to the genuine paths of science. It will be to him a felicity that will soften the toil, and lighten the labor, if he *can* rescue individual merit from obscurity, place the aggregate excellencies of his countrymen in a conspicuous view, and raise the medical character of the empire to its proper station in the ranks of science. But here it may be allowed, without imputed affectation, to pause on the execution of so vast a design; to contemplate, with almost fearful apprehension, the wild chaos of jarring opinions that bewilder the imagination, and disturb the judgment; or to shrink from the abyss of research, inevitably connected with the prosecution of the work.

From among the multitude of books that present for examination, it will be an arduous task to discriminate and select; to award to each its due portion of attention, to explain their opinions, and state their facts; to ascertain the discoveries they claim, and elucidate the theories they support. But a still more difficult, delicate, and dangerous point it will be, to appreciate merit; to touch on defects without offensive criticism; to apportion praise and censure, without raising resentment. Fully sensible of the impossibility of altogether avoiding observations that may give offence, the author is solicitous to deprecate wrath, by asserting the spirit of candour and truth, by which he will anxiously endeavour to govern his animadversions. When he is compelled, by the nature of his plan, to speak of the works of writers, still living, he hopes to be believed, when he affirms that the narrowness of party, the preference of system, or the attachment to a school, will not influence his remarks: that when he differs in opinion, as at times he must, he trusts it will be done without asperity; and that when he attempts to argue, it will not be for *victory*, but for TRUTH.

